# Frequency-resolved optical gating technique for retrieving the amplitude of a vibrational wavepacket

**DOI:** 10.1038/srep11366

**Published:** 2015-06-12

**Authors:** Yasuo Nabekawa, Yusuke Furukawa, Tomoya Okino, A. Amani Eilanlou, Eiji J. Takahashi, Kaoru Yamanouchi, Katsumi Midorikawa

**Affiliations:** 1 Attosecond Science Research Team, RIKEN Center for Advanced Photonics (RAP), 2-1 Hirosawa, Wako-shi, Saitama 351-0198, Japan; 2 Department of Chemistry, School of Science, The University of Tokyo, 7-3-1 Hongo, Bunkyo, Tokyo 113-0033, Japan

## Abstract

We propose a novel method to determine the complex amplitude of each eigenfunction composing a vibrational wavepacket of 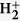/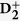 molecular ions evolving with a ~10 fs time scale. We find that the two-dimensional spectrogram of the kinetic energy release (KER) of H^+^/D^+^ fragments plotted against the time delay of the probe pulse is equivalent to the spectrogram used in the frequency-resolved optical gating (FROG) technique to retrieve the complex amplitude of an ultrashort optical pulse. By adapting the FROG algorithm to the delay-KER spectrogram of the vibrational wavepacket, we have successfully reconstructed the complex amplitude. The deterioration in retrieval accuracy caused by the bandpass filter required to process actual experimental data is also discussed.

The coherent ultrafast dynamics of matter is governed by the coherent superposition of multiple quantum states described with wavefunctions, which we call a wavepacket. We can find many studies that deal with the real-time evolution of a wavepacket composed of various quantum states, such as electronic bound states in an atom^1^,^2^,^3^,^4^, continuum electronic states with the ionization of an atom^5^, and rotational^6^ and vibrational^7^,^8^ states of a molecule. This is due to the fact that the wavepacket exhibits the fundamental characteristics of quantum mechanics^9^,^10^,^11^, and thus has fascinated many researchers. In addition, the wavepacket is a strong candidate system for manipulating quantum information^12^.

As a specific target for investigating fundamental physics in a molecule, the vibrational wavepacket of 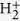/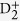
13,14,15,16,17,18 has strongly attracted our interest because 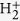/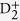 is the simplest molecular system composed of only two nuclei and one electron. The ultrashort vibrational period with 10 fs order is another feature attractive to the community of ultrafast optical science.

The common basic scheme for observing the wavepacket dynamics follows the conventional pump-probe measurement, in which the pump pulse ionizes a neutral H_2_/D_2_ molecule to create a vibrational wavepacket, and then, after a delay, the probe pulse is irradiated to dissociate a 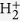/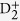 molecular ion. The spectrogram of the kinetic energy release (KER) determined from the observed H^+^/D^+^ fragment ions with the scanning delay of the probe pulse reveals the vibrational motion of the wavepacket. The most significant and basic requirement for the pump and probe pulses is that both pulse durations should be much shorter than the vibrational period. Ergler *et al.*^14^ successfully observed the vibrational motion by using a pair of intense sub-10-fs pulses of a Ti:sapphire laser as the pump and probe pulses. Kelkensberg *et al.*^16^ demonstrated that an isolated attosecond pulse generated as a high-harmonic field of an a-few-cycle Ti:sapphire laser pulse is useful for generating the vibrational wavepacket of 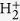, the time evolution of which was revealed with another a-few-cycle Ti:sapphire laser pulse. We have also shown^18^ that an attosecond pulse train (APT), which was generated from a 14 fs terawatt pulse of a Ti:sapphire laser, can decompose the time evolution of vibrational states into the KER region thanks to the multiple color components in deep/vacuum ultraviolet (DUV/VUV) regions included in the sub-10-fs probe pulse.

The physical model for specifying the vibrational wavepacket of 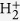/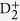 is also common in these studies^13^,^19^,^20^,^21^,^22^. The probability amplitude for finding the wavepacket at the internuclear distance *R* at time *t* is described as a coherent superposition of the vibrational states with the time-evolving phase factor:


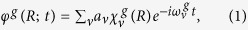


where 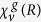 and 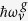 are the *ν*th vibrational eigenfunction and its eigenenergy, respectively. We denote Planck’s constant divided by *π* as *ħ*. The amplitude of each eigenfunction, *a*_*ν*_, which determines the wavepacket function at the initial time, should be found by considering all the quantum degrees of freedom in the pumping (ionization) process. Nevertheless, the overlap integral between 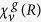 and 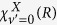 is often substituted for this amplitude by assuming the Franck-Condon principle via ionization, where 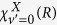 is the ground vibrational function of the H_2_/D_2_ molecule. The phase of the complex amplitude *a*_*ν*_, *arg*{*a*_*ν*_}, is fixed under this approximated condition (Franck-Condon approximation, FCA). Urbain *et al.* found that the magnitude of *a*_*ν*_ significantly deviated from that obtained under the FCA via tunneling ionization^23^. Jiang and coworkers^17^,^24^ pointed out that *a*_*ν*_ should generally be a complex number and that the non-FC model accompanying the transition of the electronic state with a continuum electron was essential for reproducing their experimental data. They still did not, however, observe *arg*{*a*_*ν*_}.

The aim of our research reported in this paper is to determine the complex amplitude, *a*_*ν*_, from experimental data so as to completely reconstruct the vibrational wavepacket of 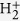/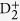. This is very similar to the determination of the Fourier amplitude of an ultrashort optical pulse at the (angular) frequency *ω*, 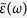, because the complex amplitude of the optical electric field, *ε*(*z*;*t*), is described as


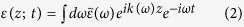


when we can approximate the optical electric field as a plane wave propagating along the *z*-axis. The plane-wave function, *e*^*ik*(*ω*)*z*^, alters with *ω* through the dispersion relation of the wavenumber, *k*(*ω*), against *ω* and gives us the functional basis set used to expand *ε*(*z*;*t*), as the vibrational wavefunction, 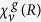, in Eq.(1) forms the functional basis for *φ*^*g*^(*R*;*t*).

Therefore, it is reasonable to consider that a technique used to retrieve the amplitude of an optical pulse may also be useful for reconstructing a vibrational wavepacket. We have developed this simple idea and found that the experimental scheme in our previous study^18^ is desirable for applying the frequency-resolved optical gating (FROG) technique^25^,^26^, which is nowadays considered a conventional technique for characterizing an ultrashort optical pulse in the range from ps^27^ to as^28^. In the following section, we briefly review our previous study in which we observed the real-time motion of the vibrational wavepacket of 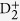, and then we show that the physical model of the probing process for the vibrational wavepacket is very similar to that for describing the FROG spectrogram. After describing the development of the FROG algorithm for the vibrational wavepacket, which we call ‘Matter-Wave FROG (MW-FROG)’, we report on the implementation of MW-FROG for a modeled vibrational wavepacket and discuss the accuracy of the retrieved data.

## Experimental scheme

**Figure 1:**
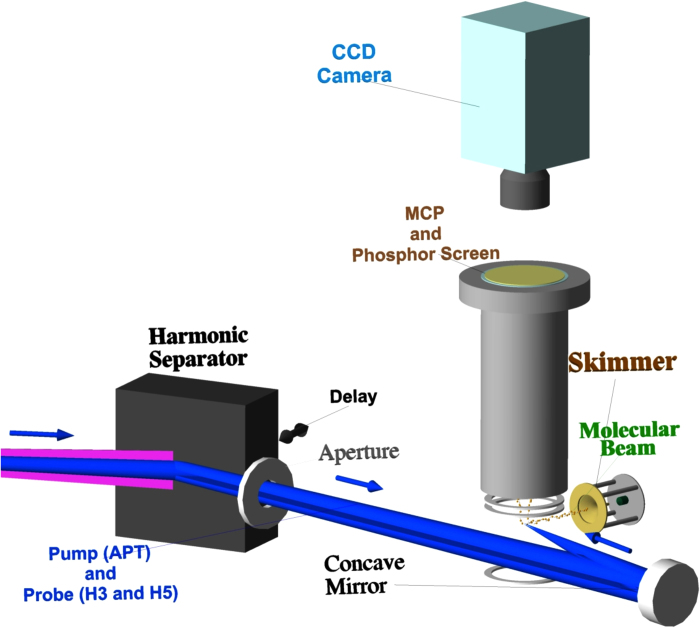
Experimental setup for observing the vibrational wavepacket of D_2_^+^ in our previous study. An attosecond pulse train (APT) with an a-few-pulse train envelope is utilized for both the pump (ionizing D_2_ molecules) and probe (one-photon excitation to the repulsive electronic state of 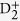) pulses.

The experimental setup for the measurement of the vibrational wavepacket of 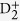 reported in ref. 18 was similar to that adopted for the measurement of the interferometric autocorrelation (IAC) signal of an APT^29^,^31^, as shown in Fig. 1. The high-order harmonic fields of a Ti:sapphire laser with a duration of 14 fs^32^ were generated from a Xe gas target in a 10-cm-long static gas cell and spatially split into two beams with a pair of silicon harmonic separator mirrors. Each harmonic separator mirror strongly attenuated the fundamental laser pulse, while the 11th- or higher-order harmonic fields, the photon energy of which exceeded the ionization energy of the H_2_ molecule, were efficiently reflected by the harmonic separator mirrors. The weak residual fundamental, 3rd-, and 5th-order harmonic components were also contained in the reflected pulse.

The twin harmonic pulses reflected from the silicon mirror pair were focused into a molecular beam of D_2_ injected through a skimmer to partition the chamber from a differential pumping section that contained a pulsed gas valve. The generated ion fragments of D^+^ were analyzed using a velocity-map-imaging (VMI) spectrometer to resolve the angular distribution and kinetic energy (KE) of the fragments. We obtained the KER spectrum for the dissociation of D^+^ + D by angularly integrating the recorded VMI with double the KE.

**Figure 2:**
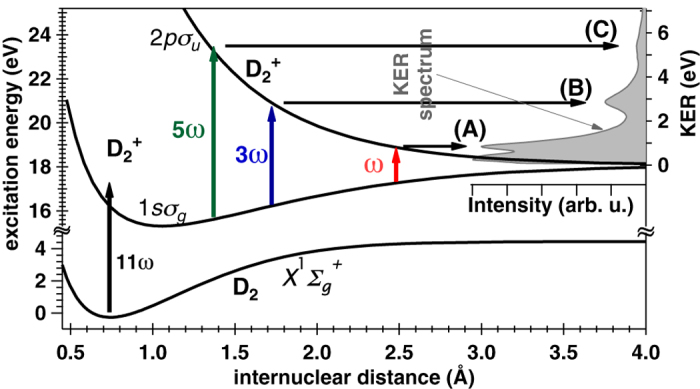
Energy diagram of the D_2_ molecular system relevant to the pump-probe measurement in our experimental scheme. The vibrational wavepacket on the adiabatic potential of the 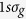 state is created by one-photon ionization by the irradiation of harmonic components in the APT, the orders of which are 11th and higher. The vibrational state is probed by excitation to the 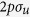 state with the one-photon absorption of the fundamental, 3rd-, and 5th-order components in the APT.

We had already specified in preceding studies^18^,^29^ that the dissociation process was accompanied by the excitation of the 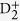 molecule from the ground bound electronic state (1*sσ*_*g*_) to the repulsive electronic state (2*pσ*_*u*_) by the one-photon absorption of the fundamental, 3rd-order, and 5th-order harmonic components. We could discriminate which order harmonic component contributed to the dissociation by resolving the KER spectrum, because the excitation is likely to occur at the nuclear distance where the energy difference between the two states is similar to the photon energy. The energy diagram of the relevant adiabatic potentials is shown in Fig. 2.

We scanned the delay between the twin harmonic pulses by translating one of the silicon harmonic separator mirrors and found that the KER spectrum is periodically modulated in accordance with the delay, as shown in Fig. 3 in ref. 18, as clear evidence of the vibrational wavepacket motion with a period of ~22 fs. This is due to the short pulse duration of the ionizing pulse, which was expected to be ~5 fs or less from the results of the IAC measurement utilizing the two-photon ionization of the N_2_ molecule^33^,^34^, and that of the probe pulse with a duration expected to be ~7 fs. We adopt a well-known theoretical model for the two-level system of the electronic state to describe the probing process used in the experiment in the next subsection.

### Theoretical model for the probe process

We assume that the initial vibrational wavepacket instantaneously appears in the ground electronic state of 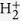 (1*sσ*_*g*_), instead of 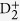, for simplicity, at *t* = 0, and then the probe optical pulse, which is composed of the coherent superposition of the 3rd- and 5th-order harmonic pulses of a Ti:sapphire laser pulse, is irradiated at a delay of *τ*, resulting in the excitation of 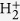 from the 1*sσ*_*g*_ state to the 2*pσ*_*u*_ state. The essential features of the vibrational states of 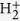 and 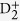 are the same. We neglect the contribution of the fundamental laser pulse because of the low signal-to-noise ratio of the beat frequency observed in the experiment^18^.

It is well known^13^,^19^,^20^,^21^,^22^ that the physical model of this system can be described with the Hamiltonian


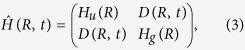


and with the electronic state


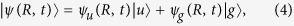


satisfying the Schrödinger equation


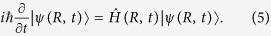


Here, *R* is the relative nuclear distance coordinate, and the vectors |*u*〉 and |*g*〉 are the eigenvectors of the electronic excited and ground states, expressed as |*u*〉 ≡ ^*T*^(1,0) and |*g*〉 ≡ ^*T*^(0,1), respectively. The Hamiltonian *H*_*u*/*g*_(*R*) describes the nuclear motion in the 2*pσ*_*u*_ and 1*sσ*_*g*_ states, and is defined as *H*_*u*/*g*_(*R*) ≡ −(2*M*)^−1^*ħ*^2^∂^2^/∂*R*^2^ + *V*_*u*/*g*_(*R*) for the reduced hydrogen mass *M* and adiabatic potential *V*_*u*/*g*_(*R*). The delayed probe laser pulse at time *t* is expressed as *E*(*t* − *τ*), and this laser pulse interacts with the electronic states via the dipole moment *μ*(*R*), which generally depends on the relative nuclear distance. Thus, *D*(*R*,*t*) in the off-diagonal elements in the Hamiltonian matrix on the right-hand side of Eq.(3) is expressed as *D*(*R*,*t*) ≡ *μ*(*R*) *E*(*t* − *τ*). The wave functions *ψ*_*u*_(*R*,*t*) and *ψ*_*g*_(*R*,*t*) give us the probability amplitudes of the nuclear motion in the 2*pσ*_*u*_ and 1*sσ*_*g*_ states, respectively.

When we already know the eigenstates of *H*_*u*/*g*_(*R*), we can regard 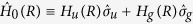 as the unperturbed Hamiltonian, and the total Hamiltonian, 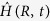, should be divided into two parts, 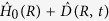, where we define the perturbing Hamiltonian as 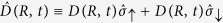. In these equations, we define the projection operator to the 2*pσ*_*u*_ (1*sσ*_*g*_) state as 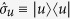 (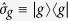) and the raising (lowering) operator from the 1*sσ*_*g*_ (2*pσ*_*u*_) state to the 2*pσ*_*u*_ (1*sσ*_*g*_) state as 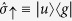 (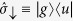).

Thus, we apply the standard recipe of time-dependent perturbation theory to Eq.(5) with the initial condition that the wavefunction, |*ψ*(*R*, *t* = 0)〉, is equal to *ψ*_0*g*_(*R*)|*g*〉, where *ψ*_0*g*_(*R*) should be the initial vibrational wavepacket,


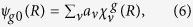


by considering the amplitude of the 2*pσ*_*u*_ state to be 0 at *t* = 0. The *ν*th vibrational wavefunction, 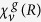, satisfies the eigenequation 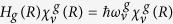, and this functional basis set satisfies the orthogonal relation 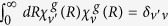.

The general solution up to the first order of the perturbation should be in the form


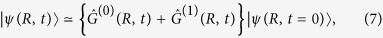


where 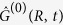 is the 0th-order expansion term describing the free propagator in each electronic state and is expressed as


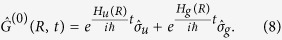


The first-order term of the propagator, 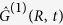, expresses the one-photon transition between the two electronic states, that is,


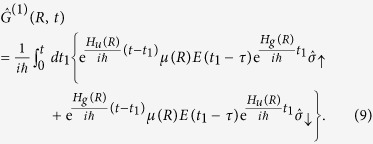


We observe, in the actual experiment, the KE spectrum of H^+^ (D^+^) from the dissociation, which should be equivalent to the KER spectrum of the dissociative H + H^+^ (D + D^+^) in the 2*pσ*_*u*_ state. Thus, we impose the final state, 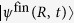, as an eigenstate of *H*_*u*_(*R*) in the 2*pσ*_*u*_ state, which is given by


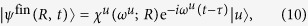


where *χ*^*u*^(*ω*^*u*^; *R*) satisfies the eigenequation *H*_*u*_(*R*)*χ*^*u*^(*ω*^*u*^; *R*) = *ħω*^*u*^*χ*^*u*^(*ω*^*u*^; *R*). The phase factor of 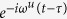 describing the time evolution of the wavefunction appears in Eq.(10) because the time interval from *τ* to *t* is *t* − *τ*. We can obtain the transition amplitude by the first-order approximation,


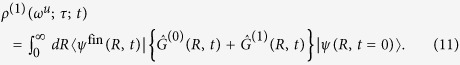


Putting the initial and final states in this equation and using the eigenequations for 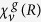 and *χ*^*u*^(*ω*^*u*^; *R*), *ρ*^(1)^(*ω*^*u*^; *τ*; *t*) should be given by


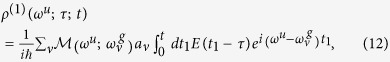


with the dipole matrix elements


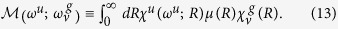


It is reasonable to assume that time *t* at the detection of H^+^ (D^+^) is infinitely large compared with the delay *τ*; thus, the upper limit of the time integral in Eq.(12) can be infinity.

We apply another approximation to Eq.(12). The magnitude of the probe pulse, |*E*(*t*)|, should be negligibly small in the time regions t ≲ – τ_p_ and τ_p_ ≲ t, where *τ*_p_ is the pulse (train envelope) width of |*E*(*t*)|. Therefore, we can approximate the time integration as


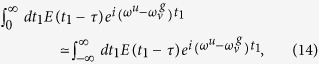


under the condition τ ≳ τ_p_ With this approximation, we obtain


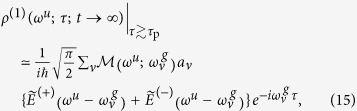


where 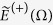 and 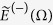 are the positive- and negative-frequency parts of the complex Fourier amplitude 
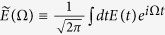
, respectively.

We neglect the negative-frequency part in Eq.(15) (rotating wave approximation) because (i) the relation 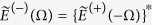 holds for the real optical field of *E*(*t*), (ii) the magnitude of 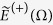 should be finite only in the frequency region around Ω~*ω*_3_ > 0 and Ω~*ω*_5_ > 0 and 0 otherwise, and (iii) the argument of the Fourier amplitude 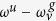 is a positive number for all eigenenergies of *ħω*^*u*^ and 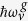.

By removing the overall constant factor from the right-hand side in Eq.(15) and replacing 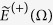 with 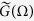, which is equivalent to the Fourier amplitude of the complex optical field of the probe (gate) pulse, we obtain the transition amplitude, *T*(*ω*^*u*^;*τ*), as


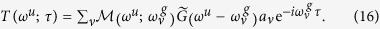


We can easily recognize the similarity between the transition amplitude, *T*(*ω*^*u*^;*τ*), in Eq.(16) and the FROG amplitude obtained by the equation


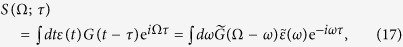


where |*S*(Ω;*τ*)|^2^ gives us the frequency-delay spectrogram of the correlation signal of an optical field of *ε*(*t*) and the gate field *G*(*t* − *τ*), by considering the correspondence between 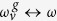, ∑_*ν*_ ↔ ∫*dω*, 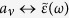, and *ω*^*u*^ ↔ Ω. Although we find the difference of *T*(*ω*^*u*^;*τ*) from *S*(Ω;*τ*) by 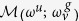, we can obtain this matrix element with a modeled theoretical calculation based on the spectroscopic data. Thus, we conclude that the FROG algorithm can be applied to *T*(*ω*^*u*^; *τ*) provided that we give the theoretical result of 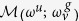 to the algorithm.

**Figure 3:**
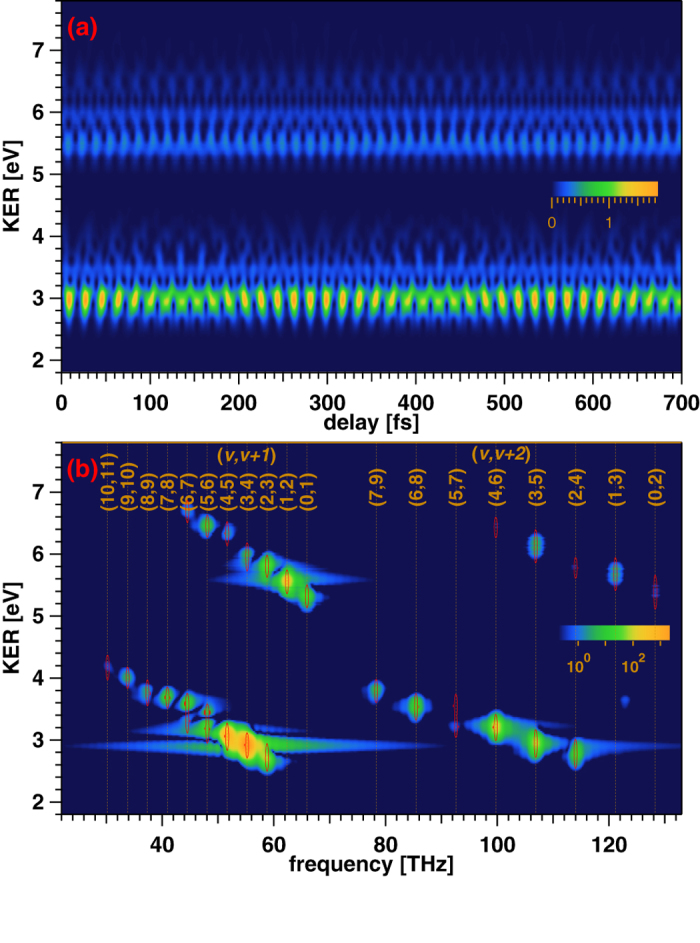
(**a**) Delay-KER spectrogram calculated from |*T*(*ω*^*u*^;*τ*)|^2^ in Eq.(16) by assuming that *a*_*ν*_ coincides with the overlap integral and that the gate pulse is the Fourier-limited pulse obtained from the measured spectra of the 3rd- and 5th-order harmonic fields. (**b**) Magnitude square of the Fourier transform of the delay-KER spectrogram depicted in Fig. 3(a). The color scale used to plot the intensity is logarithmic. The bandpass filter applied to the test target image depicted in the first panel in the last figure of this paper is also shown as contour plots.

We show the delay-KER spectrogram calculated from |*T*( *ω*^*u*^;*τ*)|^2^ in Fig. 3(a). We substitute 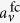 for *a*_*ν*_, where 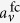 is the conventional overlap integral under the FCA condition in this calculation. We put the square root of the measured profile of the spectrum of the probe pulse, which is obtained by the superposition of the measured spectra of the 3rd- and 5th-order harmonic fields, into the magnitude profile of the gate field, 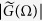. We also assume a flat spectral phase of 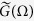. The vibrational structure lies in two separate KER regions, one of which is around ~3 eV and the other is around ~5.6 eV. The 3rd- and 5th-harmonic components in the gate field contribute to the formation of the structure in the former and latter KER regions, respectively.

Note that we cannot distinguish an arbitrary delay of the gate field from an advance of the wavepacket by observing |*T*(*ω*^*u*^;*τ*)|^2^. When the gate field includes an additional delay, *τ*^G^, the transition amplitude, *T*(*ω*^*u*^;*τ*), is modified to *T*^G^(*ω*^*u*^;*τ*) such that


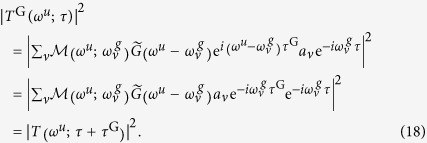


We find that 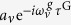 is the wavepacket amplitude that has already evolved with time *τ*^G^. This group delay (GD) may also be an unintentional offset of the delay origin; thus, we can eliminate an arbitrary phase proportional to the binding energy from the retrieved phase of *a*_*ν*_ to find a nontrivial phase modulation.

The magnitude square of the Fourier transform of |*T*(*ω*^*u*^;*τ*)|^2^ shown in Fig. 3(b) exhibits the beat frequency components 
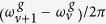
 and 
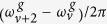
, which are spectrally resolved along the KER direction. This is due to the fact that the gate field, 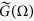, actually performs a ‘gate’ action by bandpass filtering for *ω*^*u*^ so as to satisfy the condition 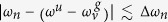 (*n* = 3, 5), where *ω*_*n*_ and Δ*ω*_*n*_ are the peak frequency and bandwidth of the *n*th harmonic component, respectively. The highest vibrational number that appears as a beat frequency is 9 or 10. Thus, we do not expect the reconstruction of the wavepacket amplitudes of these vibrational states and higher states from the delay-KER spectrogram. Although the gate action restricts the range of beat frequency components that can be observed, it is essential to retrieve the amplitude of the wavepacket by using the FROG algorithm. We show the numerical values of beat frequencies that appear in Fig. 3(b) in Table 1. These values do not exhibit significant differences from the beat frequencies calculated from the vibrational energies given in ref. 35, and hence we conclude that our MW-FROG software code can correctly reproduce the vibrational period in the frequency-KER spectrogram. The frequency-KER spectrogram in Fig. 3(b) can be used to calibrate the delay and KER of the experimental data and to generate the bandpass filter mentioned in the following subsections.

### MW-FROG algorithm

**Figure 4:**
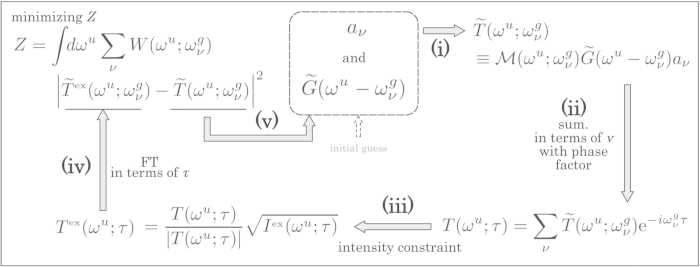
Retrieval algorithm of MW-FROG.

We define *I*^ex^(*ω*^*u*^;*τ*) as the experimental data of a delay-KER spectrogram and 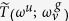 as


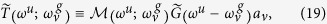


to simplify the expressions. The amplitude of the delay-KER spectrogram described in Eq.(16) is expressed as


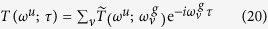


with this notation. The retrieval algorithm that we have adopted is the same as the conventional FROG algorithm using a generalized projection method to optimize the wavepacket (*a*_*ν*_) and gate-field amplitudes (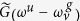) in the frequency domain, as shown schematically in Fig. 4. The initial guesses for 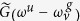 and *a*_*ν*_ generate the frequency-KER amplitude, 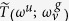, by multiplying the projection matrix 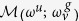 by the product of these two quantities in operation (i) in this figure. The frequency-KER amplitude is converted to a delay-KER amplitude, *T*(*ω*^*u*^;*τ*), by accumulating 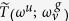 in terms of *ν* with the phase factor 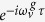 ((ii)). We obtained the experimental delay-KER amplitude, *T*^ex^(*ω*^*u*^;*τ*), by substituting (*I*^ex^(*ω*^*u*^;*τ*))^1/2^ for |*T*(*ω*^*u*^;*τ*)|, such that


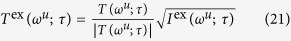


in operation (iii). We apply a Fourier transform to *T*^ex^(*ω*^*u*^;*τ*) and choose the frequency components at 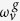 to obtain 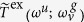 ((iv)). The next guesses for *a*_*ν*_ and 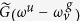 for the second loop of iterations are determined by minimizing the functional distance *Z* between 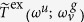 and 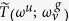 ((v)) defined as


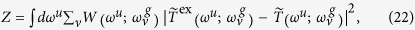


where 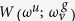 is the weight function used to ensure the appropriate convergence in iterations. Note that we do not perform the singular value decomposition of 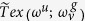 to determine *a*_*ν*_ and 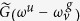 as principal components^25^ because the number of *ν* (vibrational states) is much smaller than the number of discretized *ω*^*u*^ and the 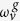 are unequally spaced in the frequency axis. We instead sequentially determine *a*_*ν*_ and 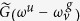 such that the functional derivative of *Z* with respect to *a*_*ν*_ or 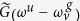 should be 0. We can also limit the parameters to be optimized in the functional space; for example, |*a*_*ν*_| can be optimized with a fixed *arg*{*a*_*ν*_}. The convergence of the iterative loop is monitored by the normalized functional difference defined as Δ_rms_ ≡ (*Z*/*Z*^ex^)^1/2^, where *Z*^ex^ is the area of 
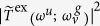
 weighted with 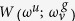.

In the actual optimization of *a*_*ν*_ and 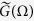, we implement the following procedures to make the unintentional GD in *a*_*ν*_, which is complementary to the advance of 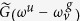 as stated in the explanation for Eq.(18), as small as possible. First, we substitute 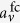 into *a*_*ν*_ and then optimize the heights of the two peaks in the gate field while the magnitude profiles are fixed to the measured spectral magnitudes of the 3rd- and 5th-order harmonic fields. The spectral phase of the gate field is also fixed to 0 in this process. Second, we optimize |*a*_*ν*_| under the condition of the fixed gate and the 0-phase of *a*_*ν*_. Third, the gate field is optimized under the initial condition of the noise field. An unintentional GD should be mainly imposed on the GD of the gate field in this process because the phase of *a*_*ν*_ is fixed to 0. After the third process, we repeat the optimization of *a*_*ν*_ and 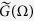 sequentially, and finally, we optimize the polynomial coefficients describing the phase of *a*_*ν*_. The reason for performing the last process will be given later.

**Figure 5:**
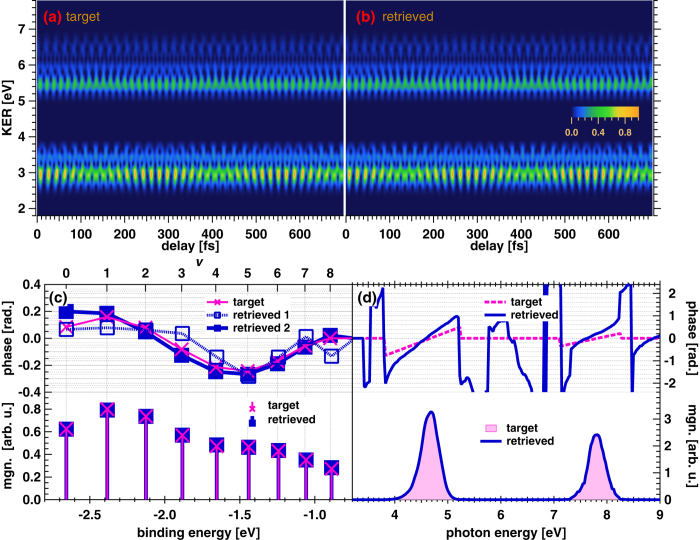
(**a**) Delay-KER spectrogram used as a target for retrieval in MW-FROG algorithm. This spectrogram is generated from the modeled wavepacket amplitude and gate field, the magnitudes of which are depicted as crosses with bars in the bottom panel of Fig. 5(c) and as a shaded area in the bottom panel of Fig. 5(d), respectively. The phases for these quantities are also shown as crosses with connecting lines in the top panel of Fig. 5(c) and as a dashed curve in the top panel of Fig. 5(d). (**b**) Delay-KER spectrogram retrieved from that in Fig.5(a). (**c**) Magnitude and phase of the wavepacket amplitude, *a*_*ν*_. The magnitude and phase retrieved from the delay-KER spectrogram are shown as solid squares with bars (magnitude) and connecting lines (phase) in the bottom and top panels, respectively. The phases depicted as hollow squares are obtained by optimization of *a*_*ν*_, while those depicted by solid squares are obtained by optimization of the polynomial expansion coefficients of the phases. (**d**) Magnitude and phase of the gate field 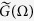. Solid curves in the bottom and top panels are the retrieved magnitude and phase, respectively.

In order to determine whether our software code implementing the MW-FROG algorithm can accurately reproduce a known target spectrogram, we generate a target spectrogram, as shown in Fig. 5(a), in accordance with a model wavepacket amplitude and gate field, the magnitude and phase of which are depicted in Fig. 5(c,d), respectively. In this model calculation, the magnitude of *a*_*ν*_ is similar to that obtained in a theoretical model of ionization with the one-photon absorption of an APT. The phase of *a*_*ν*_ is modulated with the 5th-order polynomial with respect to the binding energy. The modulation depth is approximately 0.2 rad. The target amplitude of *a*_*ν*_ for all *ν* is determined in this manner, while we set *a*_*ν*_ to zero for *ν* > 8 in the retrieved result because of the missing beat frequencies for this range of vibrational numbers in the spectrogram. The magnitude of the gate field coincides with the magnitude profile composed of the measured 3rd- and 5th-order harmonic components. The group delays of the 3rd- and 5th-order harmonic components are set to 0.9 rad/eV (
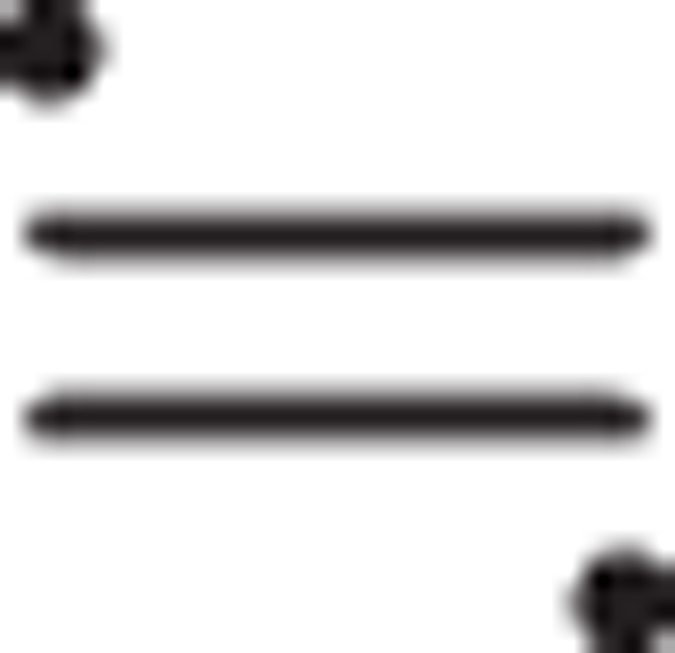
 592 as) and 0.5 rad/eV ( 263 as), respectively.

By setting the target delay-KER spectrogram to that obtained by the exact calculation of |*T*(*ω*^*u*^;*τ*)|^2^, we retrieve the delay-KER spectrogram shown in Fig. 5(b). The retrieved magnitude and phase of *a*_*ν*_ are shown as squares with bars and squares with connecting lines in the bottom and top panels in Fig. 5(c), respectively. The deviation (root mean square of differences) of the magnitude of the retrieved *a*_*ν*_ from that of the target *a*_*ν*_ is estimated to be only 6.5 × 10^−3^ on the arbitrary unit scale of |*a*_*ν*_| depicted in the bottom panel of Fig. 5(c). This result ensures the reliability of our MW-FROG algorithm. Nevertheless, it should be noted that the wavepacket magnitude should always include the deviation from the actual magnitude obtained from experimental data because we use an approximated formula (Eq.(13)) to obtain 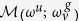 in Eq.(16).

The phase of *a*_*ν*_, labeled ‘retrieved 1’ in the top panel in Fig. 5(c), is derived by optimization of *a*_*ν*_. We corrected the GD so as to minimize the deviation from the target phase. The resultant phase shows scattering from the target phase with an r.m.s. error of 56 mrad. This is due to the processes in the MW-FROG algorithm labeled (ii) and (iv) in Fig. 4. The operation of 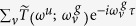 in process (ii) is not exactly the same as the inverse Fourier transform of 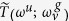 because of the unharmonicity of the vibrational frequencies, 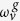. Nevertheless, we execute the Fourier transform of *T*^ex^(*ω*^*u*^;*τ*) in process (iv) as the inverse operation in process (ii), then obtain the Fourier amplitude of 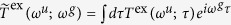 by assuming that *ω*^*g*^ is a continuos variable. In the actual calculation in this process, the variables *τ* and *ω*^*g*^ are discretized with equal steps of Δ*ω*^*g*^ and Δ*τ*, respectively, where these two quantities satisfy the equation Δ*ω*^*g*^ = 2*π*/(*N*Δ*τ*), where *N* is the number of points. We replace *ω*^*g*^ by 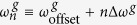 (*n* = 0,1,…,*N* − 1) and *τ* by *τ*_*m *_≡ *τ*_offset_ + *m*Δ*τ* (*m* = 0,1,…,*N* − 1). The Fourier amplitude is expressed as 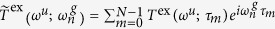. Therefore, we have to always choose the nearest-neighbor frequency, 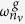, as the approximated 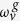 and regard 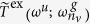 as 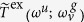. This situation is graphically explained in FIG. S-1 of the supplementary information. The deviation of 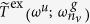 from 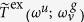 in the algorithm is the source of the scatter in the retrieved phase of *a*_*ν*_.

We have confirmed that the scatter is significantly reduced by zero padding to increase the number of points, *N*, before the Fourier transform because of the reduced frequency step, Δ*ω*^*g*^. The results are presented in FIG. S-2 of the supplementary information. Currently, the maximum available *N* is 2^16^, which is limited by the 4 GB memory size in the 32-bit architecture of our personal computer software, resulting in the phase error of 56 mrad mentioned above. The functional error, Δ_rms_, is 3.1%. In FIG. S-5 of the supplementary information, we also show another test of the performance of the MW-FROG algorithm using a different target *a*_*ν*_ and 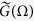, resulting in a phase error of 73 mrad.

Although this accuracy is sufficient to detect a phase modulation with a magnitude of 0.2 rad, even a small amount of scattering can cause deviation in the differentiation of the phase with respect to the frequency, which is regarded as a GD modulation possibly contained in the measured spectrogram. Thus, we expand the phase into a polynomial in terms of 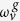 to smoothly connect the adjacent phases and optimize the coefficients in the MW-FROG algorithm. The resultant phase using the optimized polynomial coefficients is shown as ‘retrieved 2’ in the top panel of Fig. 5(c). Although Δ_rms_ somewhat increases to 3.2%, the phase deviation is reduced to 47 mrad.

We note that the phase of the wavepacket amplitude should be accurately obtained from the experimental data even though we use the approximated formula of 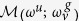 in the MW-FROG algorithm. This is because the phase information is only contained in the phases at the beat frequency components in the Fourier domain, which should not be affected by the deviation of a real function of 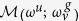. Hence, there may be another possible analytical method to extract the phase of the wavepacket from the relative phases in the beat frequencies if we can determine the characteristic of the gate field by a separate measurement. The robustness for phase retrieval under worse situations that mimic experiments is shown in the next section and the supplementary information.

The retrieved gate field is depicted in Fig. 5(d). The magnitude of the retrieved gate field coincides with that of the target field. The retrieved phase is also similar to that of the target except for the GD offset. Although the GD difference between the 3rd- and 5th-order harmonic components is estimated to be 0.26 rad/eV (171 as) larger than that of the target (0.4 rad/eV = (0.9 −0.5) rad/eV), the resultant GD difference does not significantly change the temporal profile of the gate field.

### Toward application to experimental data

To apply the MW-FROG algorithm to the experimental data, we have to resolve some issues. One is the short scanning range of the delay. The beat frequency components among the vibrational states must be clearly distinguished from each other by the Fourier transform of the delay-KER spectrogram against the delay axis so as to execute the MW-FROG algorithm correctly. The frequency resolution required is approximately 3 THz, and thus, the scanning range of the delay should be much longer than 300 fs, while the scanning range of our experimental data in the previous study^18^ is only ~140 fs. We can, however, easily extend the scanning range by replacing the translation stage used to adjust the position of the silicon harmonic separator mirror, and we expect a delay range of more than 500 fs with this modification.

Another issue may arise from the noise inherent in the experiment. This is common in conventional FROG measurements of ultrashort optical pulses, which can be resolved using a method similar to that adopted in the FROG measurement. High-frequency noise far from the relevant frequency range of the measured optical pulse is rejected using a low-pass filter before implementing the FROG algorithm^25^. We will also need a filter to eliminate the frequency components unnecessary for determining the wavepacket amplitude from the target spectrogram in our MW-FROG measurement. For this purpose, it is appropriate to apply a bandpass filter to the target delay-KER spectrogram obtained with the experimental data because the discrete frequency peaks of the Fourier transform of the spectrogram should be fixed to the beat frequencies of the vibration, unlike the continuum frequency spectrum of the Fourier transform of the FROG spectrogram for an isolated ultrashort optical pulse, as shown in Fig. 3(b).

In order to examine the performance of the MW-FROG algorithm with a filtered target spectrogram, we determined the transmission band of the bandpass filter, shown as contours in Fig. 3(b), by extracting the KER profile at each beat frequency in Fig. 3(b). The beat notes whose number difference is greater than 2 are omitted. The positions of KER peaks in the bandpass filter are adjusted to coincide with those in Fig. 3(b). The KER width of the filter, which is fit to a Gaussian profile, is set to twice that in Fig. 3(b). The window function for the frequency is the *δ*-function with a finite number of points, *N*, that is, sin((*N* + 1)(*ω* − *ω*_peak_)Δ*τ*/2)/sin((*ω* − *ω*_peak_)Δ*τ*/2), where Δ*τ* is the time step for the delay increment. *N* is set to twice the number of points used for delay scanning to reduce the endpoint effects.

Before Fourier transforming the spectrogram to apply the bandpass filter, we remove the DC component from the spectrogram to eliminate the background tail (ringing) of the high-DC component in the beat frequency range. After applying the bandpass filter, we add a DC component as the delta function with the average KER profile such that the intensity of the spectrogram after the inverse Fourier transform is greater than or equal to zero. Unintentional negative parts in the spectrogram are truncated.

**Figure 6:**
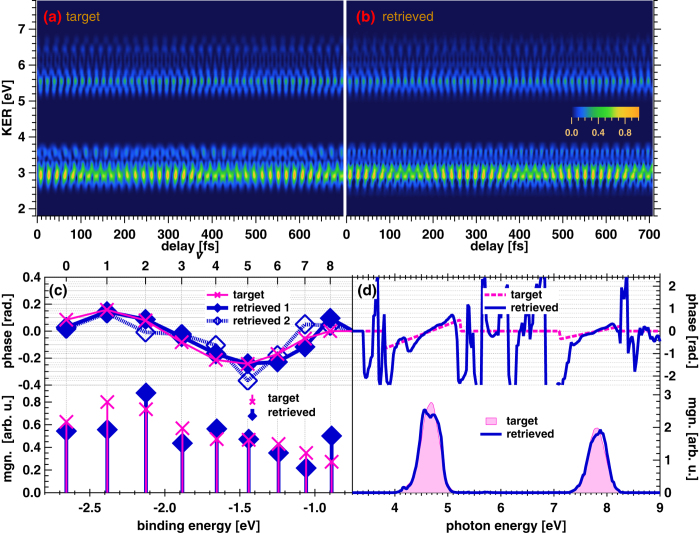
(**a**) Target delay-KER spectrogram after passing through the bandpass filter, which is depicted as contour plots in Fig.3. The modeled wavepacket amplitudes and gate field are the same as those used to generate the delay-KER spectrogram in Fig. 5(a). (**b**) Delay-KER spectrogram retrieved from that in Fig. 6(a). (**c**) Magnitude and phase of the wavepacket amplitude, *a*_*ν*_. The magnitude and phase retrieved from the delay-KER spectrogram are shown as solid diamonds with bars (magnitude) and connecting lines (phase) in the bottom and top panels, respectively. The phases depicted as hollow diamonds are obtained by optimization of *a*_*ν*_, while those depicted by solid diamonds are obtained by optimization of the polynomial expansion coefficients of the phases. (**d**) Magnitude and phase of the gate field 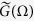. Solid curves in the bottom and top panels are the retrieved magnitude and phase, respectively.

We obtained the target delay-KER spectrogram by applying the bandpass filter, as shown in Fig. 6(a), then checked the accuracy of the retrieved *a*_*ν*_ and 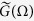. The resultant delay-KER spectrogram is shown in Fig.6(b), and the magnitude and phase of *a*_*ν*_ are depicted as diamonds with bars and diamonds with connecting lines in the bottom and top panels of Fig. 6(c), respectively. The retrieved magnitude is scattered from the target magnitude with a deviation of 0.15 on the arbitrary unit scale of |*a*_*ν*_| depicted in the bottom panel of Fig. 6(c), which is sufficiently large to disturb the observation of the change in magnitude of the target *a*_*ν*_. This is due to the fact that the bandpass filter accompanied by DC compensation disturbs the intensity profile of the target spectrogram. The functional error is increased to 18.3% (with the optimization of *a*_*ν*_) or 18.7% (with the optimization of the polynomial coefficients expressing the phase of *a*_*ν*_).

In spite of the visible deviation of the retrieved magnitude, the retrieved phase is not significantly affected by the bandpass filter, as shown in the top panel of Fig. 6(c). The phase of *a*_*ν*_ does not depend on the magnitudes of beat frequencies nor that of the DC component. To retrieve the phase of *a*_*ν*_, we only require the phase at each beat frequency. This is the reason why we can successfully retrieve the phase of *a*_*ν*_. The r.m.s. error between the target and retrieved phases is sufficiently small and is estimated to be 79 mrad. (with the optimization of *a*_*ν*_) or 51 mrad. (with the optimization of the polynomial coefficients expressing the phase of *a*_*ν*_).

The retrieved gate field, depicted in Fig. 6(d), exhibits somewhat degraded profiles in both the magnitude and phase compared with the target gate field, whereas the discrepancy is negligibly small. The GD difference between the 3rd- and 5th-order harmonic components in the retrieved gate field deviates by only 0.11 rad/eV (74 as) from that in the target gate field.

In order to simulate an experimental situation, we further examined the retrieval of the wavepacket amplitude from target delay-KER spectrograms, which were intentionally deteriorated under the following conditions. (i) The KER resolution of our VMI spectrometer is 0.18 eV around the KER value of 5.6 eV. We thus generated a target delay-KER spectrogram by convolving a Gaussian KER response function with a width of 0.2 eV in full width at half maximum with the exact target spectrogram shown in Fig. 5(a). A noise was then added. (ii) We generated a delay-KER spectrogram by using a wavepacket showing non-polynomial phase modulation, which is different from the phase shown in Fig. 5(c). The gate field is the same as that shown in Fig. 5(d). We processed the generated delay-KER spectrogram in the same manner as that described for (i). (iii) We changed the spectral magnitude profile of the gate field to have double peaks in the photon energy regions of both the third and fifth harmonic components. We also applied a group-delay dispersion to the spectral phase in both the photon energy regions and added a constant phase offset in the photon energy region of the fifth harmonic component. Then, we generated a delay-KER spectrogram using this gate field and the same wavepacket as that used to generate the spectrogram in (ii). The exact spectrogram without applying the simulated experimental condition was used as the target spectrogram in the MW-FROG algorithm. (iv) We applied the same simulated experimental condition as that implemented for (i) to the exact spectrogram used in (iii) in order to generate the target spectrogram.

The retrieved spectrograms, wavepacket amplitudes, and gate fields are depicted in FIGS S-3, S-4, S-5, and S-6 in the supplementary information. We show how the phase offset in (iii) changes the temporal profile of the gate field in FIG. S-7 in the supplementary information. We confirmed that the convergence criterion of the spectrogram, *R* < 2, defined in refs. 36,37 was satisfied under our simulated experimental conditions (i), (ii), and (iv). Even though the r.m.s. errors of the retrieved phase in *a*_*ν*_ were increased under the simulated experimental conditions, they were still less than 120 mrad with the *a*_*ν*_-optimization and less than 100 mrad with the optimization of the polynomial coefficients expressing the *a*_*ν*_-phase.

As a result, we conclude that the MW-FROG algorithm is sufficiently reliable to resolve the *a*_*ν*_-phase modulation with a depth larger than ~120 mrad even when the delay-KER spectrogram is degraded to have a finite KER resolution and noise. This is due to the extraction of the relevant phase information contained in the beat frequency peaks by the bandpass filter.

### Summary and prospects

We have proposed the MW-FROG method to retrieve the vibrational wavepacket amplitude of *a*_*ν*_ generated in the ground electronic state of a 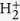/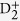 molecule based on the theoretical model of photoexcitation from the bound ground state to the repulsive excited state. The similarity of the transition amplitude in Eq.(16) to the optical FROG amplitude in Eq.(17) is due to the fact that the excitation is induced by a simple one-photon absorption process without considering the distortion of adiabatic potentials usually caused by an intense near-infrared laser pulse^15^.

We have successfully retrieved the amplitude of the vibrational wavepacket by applying the MW-FROG algorithm to the modeled target spectrogram. Although a bandpass filter, which reduce s the noise of the spectrogram obtained in an actual experiment, degraded the accuracy of the magnitude of the vibrational wavepacket, we can still accurately find a phase modulation with a modulation depth larger than ~120 mrad.

The MW-FROG method requires a priori knowledge of the nuclear wavefunctions of the system consisting of vibrational and dissociative electronic states. The scanning range of the delay should be sufficiently long to resolve each beat frequency between the vibrational states. Therefore, we may apply this method to diatomic molecules other than 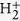 provided we obtain accurate adiabatic potential curves relevant to the system through the use of theoretical calculation and spectroscopic data. The delay-KER spectrogram originating from the vibrational motion of 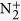 demonstrated in ref. 38 may be suitable for the application of MW-FROG if the delay range can be extended to much longer than 1.1 ps (revival time) to resolve the beat frequencies.

We have already carried out an experimental study of MW-FROG measurement for both 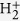 and 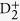 molecules by extending the scanning delay range to ~700 fs. The resultant amplitudes of the vibrational wavepackets for 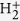 and 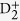 exhibit nontrivial phase modulations. Details of this study will be reported elsewhere.

## Additional Information

**How to cite this article**: Nabekawa, Y. *et al.* Frequency-resolved optical gating technique for retrieving the amplitude of a vibrational wavepacket. *Sci. Rep.* **5**, 11366; doi: 10.1038/srep11366 (2015).

## Supplementary Material

Supplementary Information

## Figures and Tables

**Table 1:** 

(***ν***,***ν***** + 1)**	**Δ*****f***_**1**_ **[THz]**	**diff. [%]**	(***ν***,***ν***** + 2)**	**Δ*****f***_**2**_ **[THz]**	**diff. [%]**
(0,1)	65.925	+0.36	(0,2)	128.28	+0.56
(1,2)	62.356	+0.78	(1,3)	121.14	+0.90
(2,3)	58.787	+1.00	(2,4)	114.01	+1.10
(3,4)	55.218	+1.10	(3,5)	106.87	+1.10
(4,5)	51.650	+1.00	(4,6)	99.730	+0.94
(5,6)	48.081	+0.82	(5,7)	92.592	+0.64
(6,7)	44.512	+0.44	(6,8)	85.455	+0.2
(7,8)	40.943	−0.07	(7,9)	78.317	−0.37
(8,9)	37.374	−0.70			
(9,10)	33.805	−1.40			
(10,11)	30.236	−2.2			

Table 1: List of beat frequencies appearing in our MW-FROG algorithm. The beat frequencies between pairs of adjacent vibrational states are denoted as Δ*f*_1_ = (*ω*_*ν*+1_ − *ω*_*ν*_)/(2*π*), and those between the next pairs of adjacent vibrational states are denoted as Δ*f*_2_ = (*ω*_*ν*+2_ − *ω*_*ν*_)/(2*π*). These numerical values are compared with those obtained from ref. 35. The resultant differences are shown as diff. in %.
