# Supplementary material for: Frequency-resolved optical gating technique for retrieving the amplitude of a vibrational wavepacket
Source: Sci Rep. 2015 Jun 12;5:11366. doi: 10.1038/srep11366 (PMC4464331; doi:10.1038/srep11366)
Supplement: Supplementary Information [file srep11366-s1.pdf]

Frequency-resolved optical gating technique for retrieving the amplitude of a vibrational wavepacket: supplementary information

Yasuo Nabekawa,[1,*] Yusuke Furukawa,[1] Tomoya Okino,[1] A. Amani Eilanlou,[1] Eiji J. Takahashi,[1] Kaoru Yamanouchi,[2] and Katsumi Midorikawa[1]

[1]*Attosecond Science Research Team, RIKEN Center for Advanced Photonics (RAP), 2-1 Hirosawa, Wako-shi, Saitama 351-0198, Japan*
[2]*Department of Chemistry, School of Science, The University of Tokyo, 7-3-1 Hongo, Bunkyo, Tokyo 113-0033, Japan*

I. FOURIER TRANSFORM IN MW-FROG ALGORITHM

As we state in the main text, the discrete Fourier transform (FT) of the MW-FROG amplitude, $T^{\rm ex}(\omega^u;\tau)$, with respect to τ in

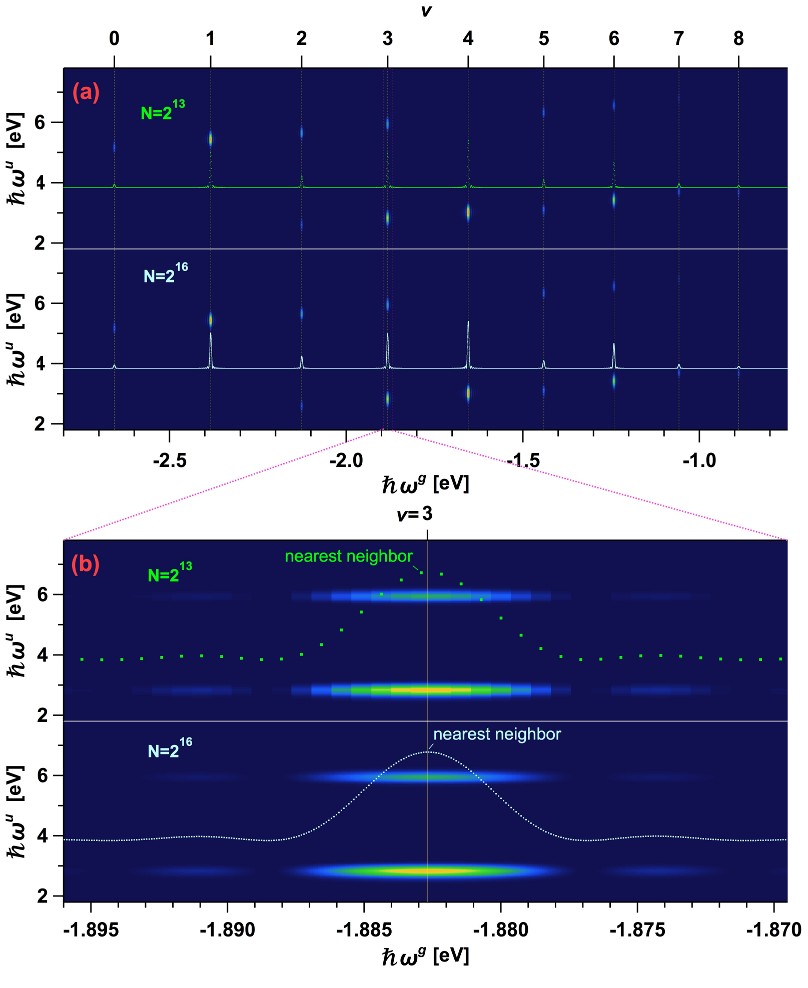

FIG. S-1. (a) $|\widetilde{T}^{\rm ex}(\omega^u;\omega^g_n)|^2$ calculated in process (iv) in the MW-FROG algorithm shown in FIG. 4 in the main text. Top panel: the number of points used for the discretization of the delay is set to 2^{13}. Bottom panel: the number of points is set to 2^{16}. The top axis indicates the vibrational numbers at the corresponding vibrational energies. The line profile obtained by integrating the image with respect to ω^u is depicted as dots in each panel. (b) Magnified views of the images and line profiles in (a) in the vicinity of a zvibrational number of 3.

* Correspondence to nabekawa@riken.jp

process (iv) in the MW-FROG algorithm intrinsically deviates from the actual $\widetilde{T}(\omega^u;\omega^g_v)$ because the vibrational frequencies, ω^g_v, are configured with unequal separations. We show that the reduction of this deviation reduces the error in the phase retrieval of the wavepacket amplitude.

In our MW-FROG algorithm, the delay τ is discretized by using a delay step of $\Delta\tau$ and a delay offset of τ_{offset} as $\tau_m = \tau_{\text{offset}} + m\Delta\tau$, where m is an integer ranging from 0 to $N_0 - 1$. The number of points used for the delay in the target spectrogram in FIG. 5(a) in the main text, N_0, is set to 2^{10} =1024. The delay step and delay offset are 0.69093984374954 fs and 0 fs, respectively. The frequency step after the discrete FT, $\Delta\omega^g/2\pi = 1/(N_0\Delta\tau)$, should be equal to 1.41338 THz, which is not sufficiently small to correctly express the beat frequencies listed in Table I in the main text. Therefore, we performed zero padding to increase the number of points N to 2^{13}, leading to $\Delta\omega^g/2\pi$ =0.176673 THz, as a first trial implementation of the MW-FROG algorithm. We notate the discretized frequency as $\omega^g_n = \omega^g_{\text{offset}} + n\Delta\omega^g$, $(n = 0, 1, \ldots, N-1)$. The resultant image of the magnitude square of $\widetilde{T}^{\text{ex}}(\omega^u;\omega^g_n)$ and its magnified view in the vicinity of a vibrational number of 3 are shown in the top panels of FIGS. S-1(a) and (b), respectively. The line profile obtained by integrating the image with respect to ω^u is also depicted as dots in the same panels. In this calculation, we use the retrieved $T^{\text{ex}}(\omega^u;\tau_m)$ obtained from the target a_v and $\widetilde{G}(\Omega)$ shown in FIGS. 5(c) and (d) in the main text. We can see from the coarse grained view that the image in the top panel of FIG. S-1(a) properly exhibits the distinct peaks of the vibrational frequencies. Nevertheless, the nearest-neighbor points from the vibrational frequencies,

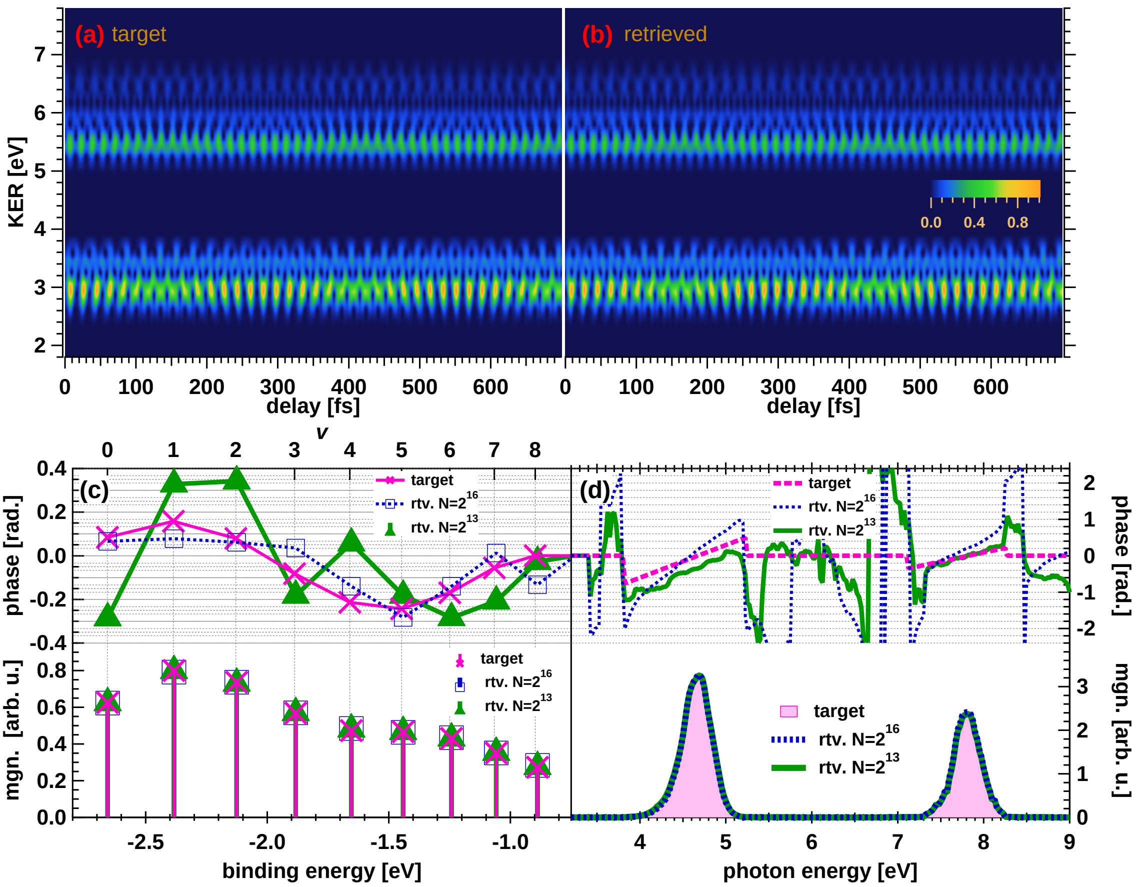

FIG. S-2. (a) Same target spectrogram as that shown in FIG. 5(a) in the main text. (b) Retrieved spectrogram with $N = 2^{13}$ points for the discrete FT in process (iv) in the MW-FROG algorithm. (c) Magnitude and phase of the wavepacket amplitude a_v. The magnitude and phase of the target are depicted as crosses with bars (magnitude) and connecting lines (phase), which are the same as those depicted in FIG. 5(c) in the main text. The retrieved magnitude and phase are shown as solid triangles with bars (magnitude) and connecting lines (phase) in the bottom and top panels, respectively. The retrieved magnitude and phase under the condition of $N = 2^{16}$ are depicted as hollow squares with bars (magnitude) and connecting dotted lines (phase) in the bottom and top panels, respectively, which are the same as those depicted in FIG. 5(c) in the main text. (d) Magnitude and phase of the gate field $\widetilde{G}(\Omega)$. The magnitude and phase of the target gate field are depicted as the shaded area in the bottom panel and the dashed curve in the top panel, respectively. The solid curves in the bottom and top panels are the retrieved magnitude and phase with the number of points of $N = 2^{13}$, respectively. The retrieved magnitude and phase under the condition of $N = 2^{16}$ are shown as dotted curves, which are the same as those depicted in FIG. 5(d) in the main text.

which are chosen as the approximated vibrational frequencies in the MW-FROG algorithm, may still differ from the vibrational frequencies by a maximum of 0.176673/2 THz. The typical deviation of the approximated vibrational frequency at $v = 3$ is graphically shown as the nearest-neighbor green dot in the top panel of FIG. S-1(b).

Under this zero-padding condition, we applied the MW-FROG algorithm to the target spectrogram depicted in FIG. 5(a) in the main text, which is the same as the target spectrogram depicted in FIG. S-2(a). The retrieved spectrogram, a_v, and $\widetilde{G}(\Omega)$ are shown in FIGS. S-2(b), (c), and (d), respectively. The retrieved magnitude of a_v, depicted as solid triangles with bars in the bottom panel of FIG. S-2(c), is almost the same as that of the target a_v, depicted as crosses with bars, with an r.m.s. error of 3.5×10^{-3} on the arbitrary unit scale of $|a_v|$. In contrast, the phase of a_v, depicted as solid triangles with connecting lines in the top panel of FIG. S-2(c), is considerably scattered from the target phase, depicted as crosses with connecting lines, resulting in an r.m.s. error of 200 mrad, which interferes with the observation of the phase modulation for the target a_v with a modulation depth of $\pm$200 mrad. The functional error Δ_{rms}, defined in the main text, is 10.7%. The retrieved magnitude and phase of the gate field, respectively depicted as solid curves in the bottom and top panels of FIG. S-2(d), are in reasonable agreement with those of the target gate field. Thus, we only needed to improve the retrieval of the phase of a_v in the MW-FROG algorithm.

This was realized by increasing the accuracy of the approximated vibrational frequencies defined in the discrete FT in process (iv). We increased the number of points, N, to 2^{16} by zero padding, and thus the frequency step after the discrete FT was reduced to 1/8 (0.022084 THz) of that adopted in the previous algorithm. The magnitude square of $\widetilde{T}^{\mathrm{ex}}(\omega^u;\omega_n^g)$ calculated under the condition of $N = 2^{16}$ and its magnified view in the vicinity of a vibrational number of 3 are shown in the bottom panels of FIGS. S-1(a) and (b), respectively. The line profile obtained by integrating the image with respect to ω^u is also depicted as dots in the same panels. We can observe from these panels that the frequency step is considerably reduced. The retrieved spectrogram, a_v, and $\widetilde{G}(\Omega)$ have already been shown in FIGS. 5(b), (c), and (d), respectively, in the main text. The retrieved magnitude and phase of a_v are also shown as hollow squares with bars (magnitude) and connecting dashed lines (phase) in the bottom and top panels in FIG. S-2(c), respectively. The scatter of the retrieved phase of a_v is sufficiently reduced to resolve the phase modulation depth of $\pm$200 mrad. for the target a_v, as we have already stated in the main text.

We note that $N = 2^{16}$ is the maximum number of points achievable using our calculation software platform (IGOR Pro 6.35A5, WaveMetrics Inc.) based on a 32-bit architecture, and the accuracy of phase retrieval may be further improved by further increasing N by using software based on a 64-bit architecture with an increased physical memory. Nevertheless, we did not attempt this because we have already obtained sufficient accuracy for the phase retrieval of a_v and we can reduce the calculation cost without using such high-performance computing. Instead, optimization of the coefficients in the polynomial expansion of the phase of a_v in the MW-FROG algorithm helped to improve the accuracy of phase retrieval as demonstrated in FIGS. 5(c) and 6(c) in the main text and also demonstrated in the next section of this supplementary information.

II. PERFORMANCE TESTS OF MW-FROG ALGORITHM

In FIGS. S-3 and S-4 in this supplementary information, we show two examples demonstrating the feasibility of our matter-wave FROG (MW-FROG) algorithm for retrieving the phases of vibrational wavepacket amplitudes using degraded delay-KER spectrograms, which simulate experimental data. We use the convergence parameter $R \equiv \varepsilon[|T_{\rm rtrvd}|^2, |T_{\rm exct}|^2]/\varepsilon[|T_{\rm exprmnt}|^2, |T_{\rm exct}|^2]$, which is defined in refs.[36] and [37] in the main text, in order to confirm the convergence criterion of $R < 2$ to be satisfied, where $\varepsilon[|T_{\rm B}|^2, |T_{\rm A}|^2]$ is the functional distance between $|T_{\rm B}(\omega^u;\tau)|^2$ and $|T_{\rm A}(\omega^u;\tau)|^2$ normalized by the square root of the average of $(|T_{\rm A}(\omega^u;\tau)|^2)^2$, namely, $\varepsilon[|T_{\rm B}|^2, |T_{\rm A}|^2] \equiv \left[\frac{1}{NM}\sum_{n=0}^{N-1}\sum_{m=0}^{M-1}(|T_{\rm B}(\omega_n^u;\tau_m)|^2 - |T_{\rm A}(\omega_n^u;\tau_m)|^2)^2\right] / \left[\frac{1}{NM}\sum_{n=0}^{N-1}\sum_{m=0}^{M-1}(|T_{\rm A}(\omega_n^u;\tau_m)|^2)^2\right]$, where ω^u and τ are discretized with the numbers of points N and M, respectively. We notate the exact spectrogram (calculated by Eq. (16) in the main text), the simulated experimental spectrogram (obtained by applying the KER convolution and noise), and the retrieved spectrogram as $|T_{\rm exct}(\omega^u;\tau)|^2$, $|T_{\rm exprmn}(\omega^u;\tau)|^2$, and $|T_{\rm rtrvd}(\omega^u;\tau)|^2$, respectively. In FIG. S-5, we demonstrate that the MW-FROG algorithm retrieves the wavepacket amplitude and gate field with similar accuracy to that obtained when we applied the MW-FROG algorithm to the target spectrogram shown in FIG. 5(a) in the main text, even when the gate field contains a chirp and a spectral phase offset. We degraded the target spectrogram in FIG S-5(a) by using the simulated experimental condition, and we show in FIG. S-6 that the MW-FROG algorithm retrieves the phases of the vibrational wavepacket amplitudes with an accuracy similar to that demonstrated for retrieving the wavepacket amplitude in FIGS. S-3 and S-4. We also show auxiliary panels to explain the alteration of the offset time of the pulse train in the train envelope by the spectral phase offset in the gate field in FIG. S-7.

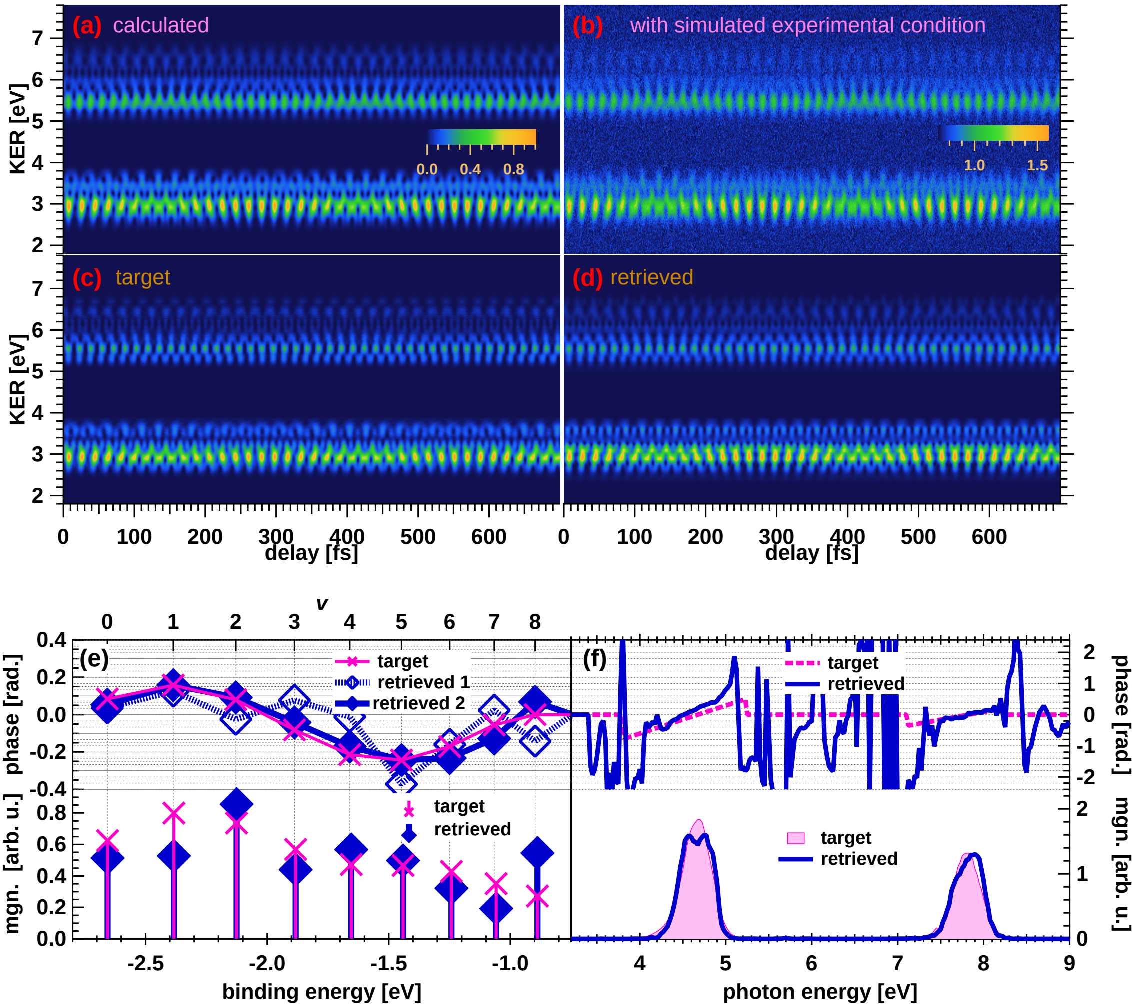

FIG. S-3. Examination of the effects of noise and degradation of the KER resolution. (a) Delay-KER spectrogram calculated from $|T(\omega^u;\tau)|^2$ in Eq. (16) in the main text, which is the same as the spectrogram shown in FIG. 5(a) in the main text. (b) We degrade the KER resolution of the delay-KER spectrogram shown in FIG. S-3(a) by convolving the Gaussian response function with a width of 0.2 eV in full width at half maximum to simulate the finite resolution of the VMI spectrometer we use. We also add a Gaussian noise to simulate experimental data more realistically, resting in FIG. S-3(b). The standard deviation of the noise magnitude is set to be approximately 10% of the peak height of the spectrogram in the KER region around 5.6 eV. (c) Target spectrogram of MW-FROG, which is obtained by bandpass filtering of the spectrogram shown in FIG. S-3(b). (d) Spectrogram retrieved with the MW-FROG algorithm. The functional error $\Delta_{\rm rms}$, defined in the main text, is 19.9%. The convergence parameter R is 0.84, which satisfies the convergence criterion. (e) Magnitude and phase of the wavepacket amplitude a_v. The magnitude and phase of the target are depicted as crosses with bars (magnitude) and connecting lines (phase), and those retrieved from the spectrogram in FIG. S-3(c) are shown as solid squares with bars (magnitude) and connecting lines (phase) in the bottom and top panels, respectively. The phases depicted as hollow squares are obtained by optimization of a_v and have an r.m.s. phase error of 117 mrad, while those depicted by solid squares are obtained by the optimization of the polynomial expansion coefficients of the phase and have an r.m.s. phase error of 45 mrad. The phase errors are comparable to those in the retrieved phases shown in FIG. 6(c) in the main text. (f) Magnitude and phase of the gate field $\widetilde{G}(\Omega)$. We depict the magnitude and phase of the target gate field as the shaded area in the bottom panel and the dashed curve in the top panel, respectively. The solid curves in the bottom and top panels are the retrieved magnitude and phase, respectively. The retrieved gate field is similar to that shown in FIG. 6(d) in the main text.

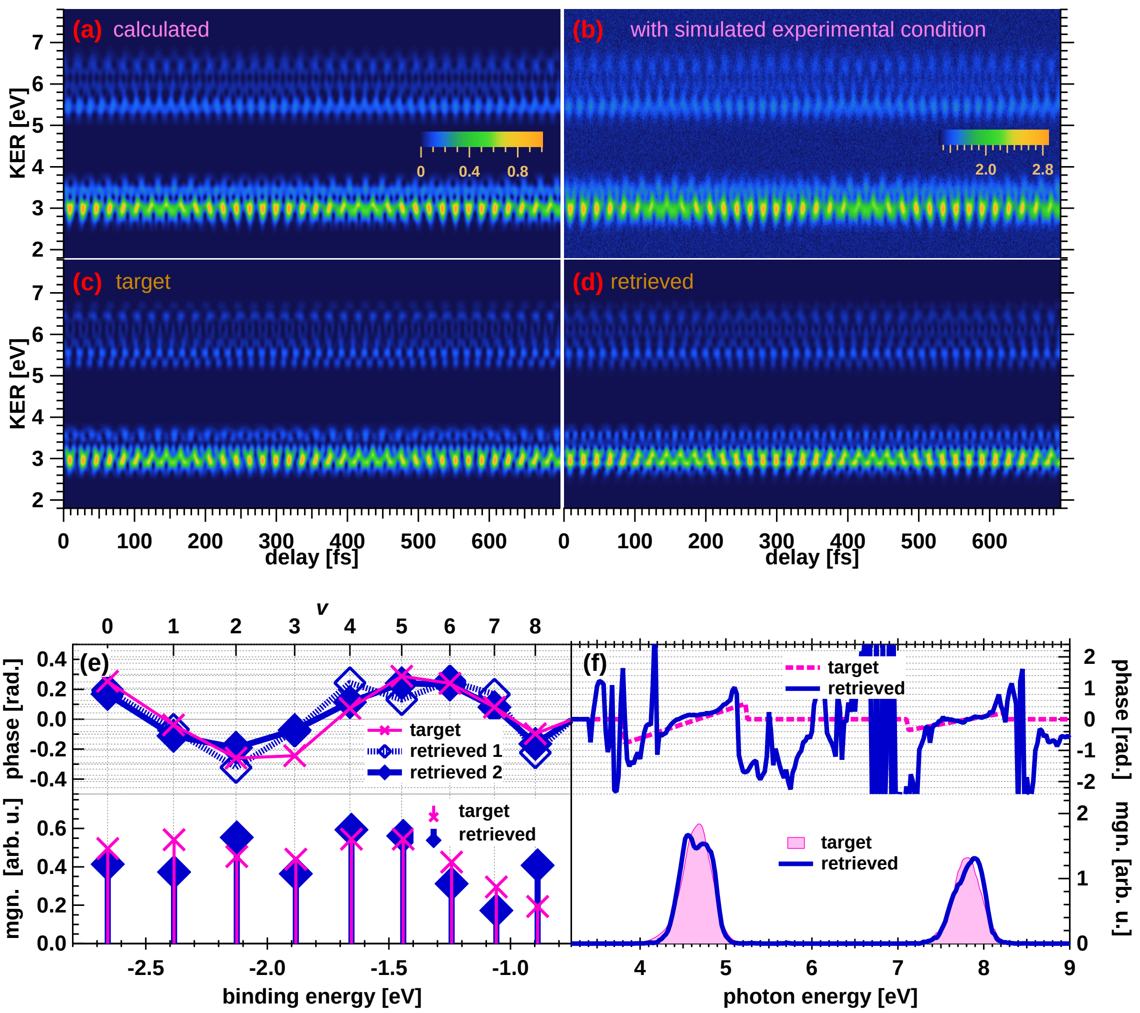

FIG. S-4. Changing the model vibrational wavepacket to one with a different magnitude and phase. The phase modulation applied to this model is generated by differentiating a Lorentzian function with respect to the binding energy. (a) Delay-KER spectrogram calculated from $|T(\omega^u;\tau)|^2$ in Eq. (16) in the main text. The model wavepacket amplitude is depicted as crosses with bars (magnitude) in the bottom panel and connecting lines (phase) in the top panel of FIG. S-4(e). (b) Spectrogram simulating experimental conditions, which is generated from the spectrogram in FIG. S-4(a) by applying the same procedures as that described in the legend of FIG. S-3(b). (c) Target spectrogram of MW-FROG, which is obtained by bandpass filtering of the spectrogram shown in FIG. S-4(b). (d) Spectrogram retrieved with the MW-FROG algorithm. The functional error $\Delta_{\rm rms}$, defined in the main text, is 19.0%. The convergence parameter R is 1.1, which satisfies the convergence criterion. (e) Magnitude and phase of the wavepacket amplitude a_v. The magnitude and phase retrieved from the spectrogram in FIG. S-4(c) are shown as solid squares with bars (magnitude) and connecting lines (phase) in the bottom and top panels, respectively. The phases depicted as hollow squares are obtained by the optimization of a_v and have an r.m.s. phase error of 112 mrad, while those depicted by solid squares are obtained by optimization of the polynomial expansion coefficients of the phase and have an r.m.s. phase error of 72 mrad. The phase errors are comparable to those in the retrieved phases shown in FIG. 6(c) in the main text. (f) Magnitude and phase of the gate field $\widetilde{G}(\Omega)$. We depict the magnitude and phase of the target gate field as the shaded area in the bottom panel and the dashed curve in the top panel, respectively. The solid curves in the bottom and top panels are the retrieved magnitude and phase, respectively. The retrieved gate field is similar to that shown in FIG. 6(d) in the main text.

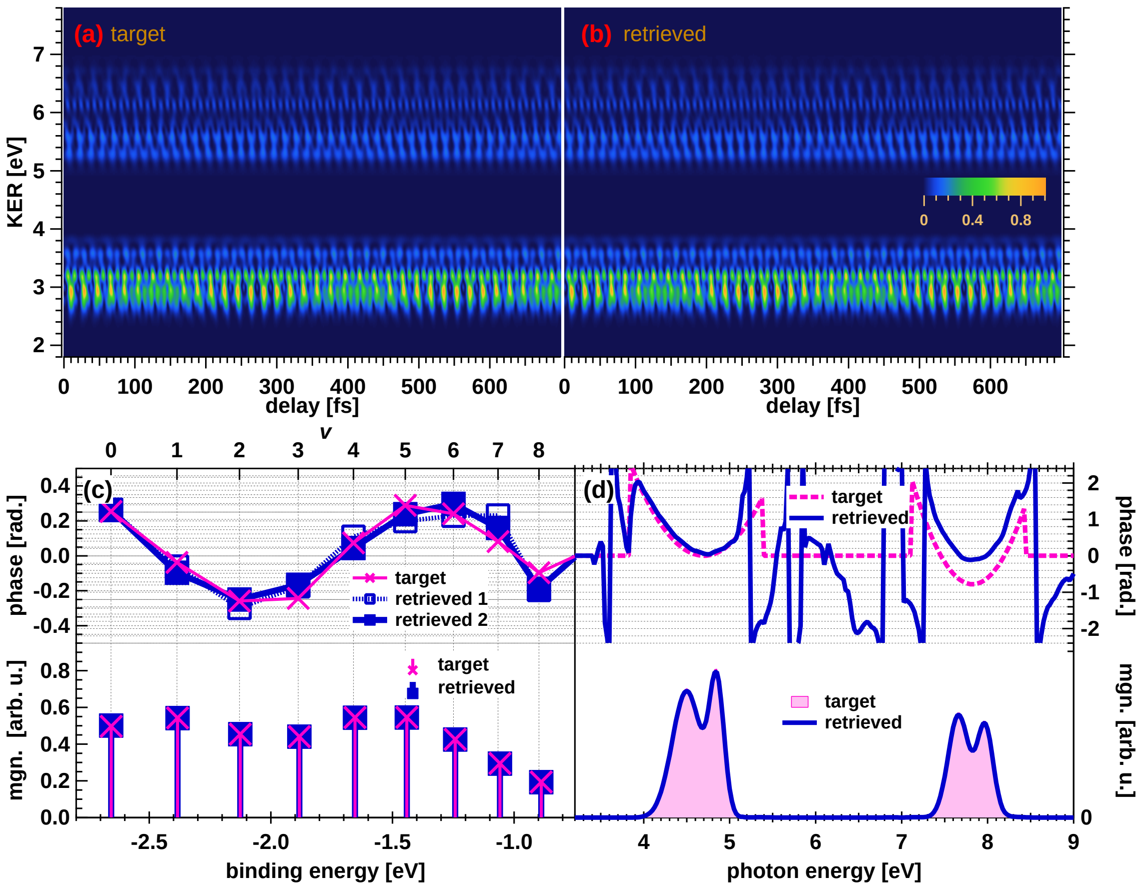

FIG. S-5. Changing the model gate field to one with group-delay dispersions (GDDs) and using the exact target spectrogram without applying noise and KER convolution. The amplitude of the model vibrational wavepacket is the same as that depicted in FIG. S-4(e). We applied GDDs of 3×10^{-30} s^2 and 5×10^{-30} s^2 in the photon energy regions of the third and fifth harmonic components, respectively, shown as a dashed curve in the top panel of FIG. S-5(d). We expect the magnitudes of both GDDs to be less than 1×10^{-29} s^2 under our actual experimental condition. A constant phase offset of $-\pi/4$ is added to the phase in the photon energy region of the fifth harmonic component. This offset phase changes the offset time of the pulse train, which is formed by the coherent superposition of the third and fifth harmonic components, in the train envelope, as shown in FIG. S-7. The magnitude of the gate field in each photon energy region is intentionally modulated to exhibit double peaks, the separation between which is set to be ∼0.3 eV, to find how the deterioration of the KER resolution affects gate magnitude retrieval. (a) Target delay-KER spectrogram calculated from $|T(\omega^u;\tau)|^2$ in Eq. (16) in the main text. The model wavepacket amplitude is depicted as crosses with bars (magnitude) in the bottom panel and connecting lines (phase) in the top panel of FIG. S-5(c). (b) Spectrogram retrieved with the MW-FROG algorithm. The functional error $\Delta_{\rm rms}$, defined in the main text, is 2.1% for a_v optimization. $\Delta_{\rm rms}$ slightly increases to 3.3% after optimization of the polynomial expansion coefficients of the phase of a_{nu}. (c) Magnitude and phase of the wavepacket amplitude a_v. The magnitude and phase retrieved from the spectrogram in FIG. S-5(a) are shown as solid squares with bars (magnitude) and connecting lines (phase) in the bottom and top panels, respectively. The phases depicted as hollow squares are obtained by the optimization of a_v and have an r.m.s. phase error of 73 mrad, while those depicted by solid squares are obtained by optimization of the polynomial expansion coefficients of the phase and have an r.m.s. phase error of 59 mrad. The phase errors are comparable to those in the retrieved phases shown in FIG. 5(c) in the main text. (d) Magnitude and phase of the gate field $\widetilde{G}(\Omega)$. We depict the magnitude and phase of the target gate field as the shaded area in the bottom panel and the dashed curve in the top panel, respectively. The solid curves in the bottom and top panels are the retrieved magnitude and phase, respectively. The magnitude of the gate field in the photon energy regions of both the third and fifth harmonic components is in good agreement with that of the target gate field. The phase of the gate field also exhibits a similar shape to that of the target field except for the constant offset of $-\pi/4$ in the photon energy region of the fifth harmonic component. The MW-FROG algorithm is insensitive to the offset time of the pulse train in the train envelope. In other words, we do not need to consider the change in the offset time of the pulse train in the train envelope to retrieve the phase of the vibrational wavepacket amplitude.

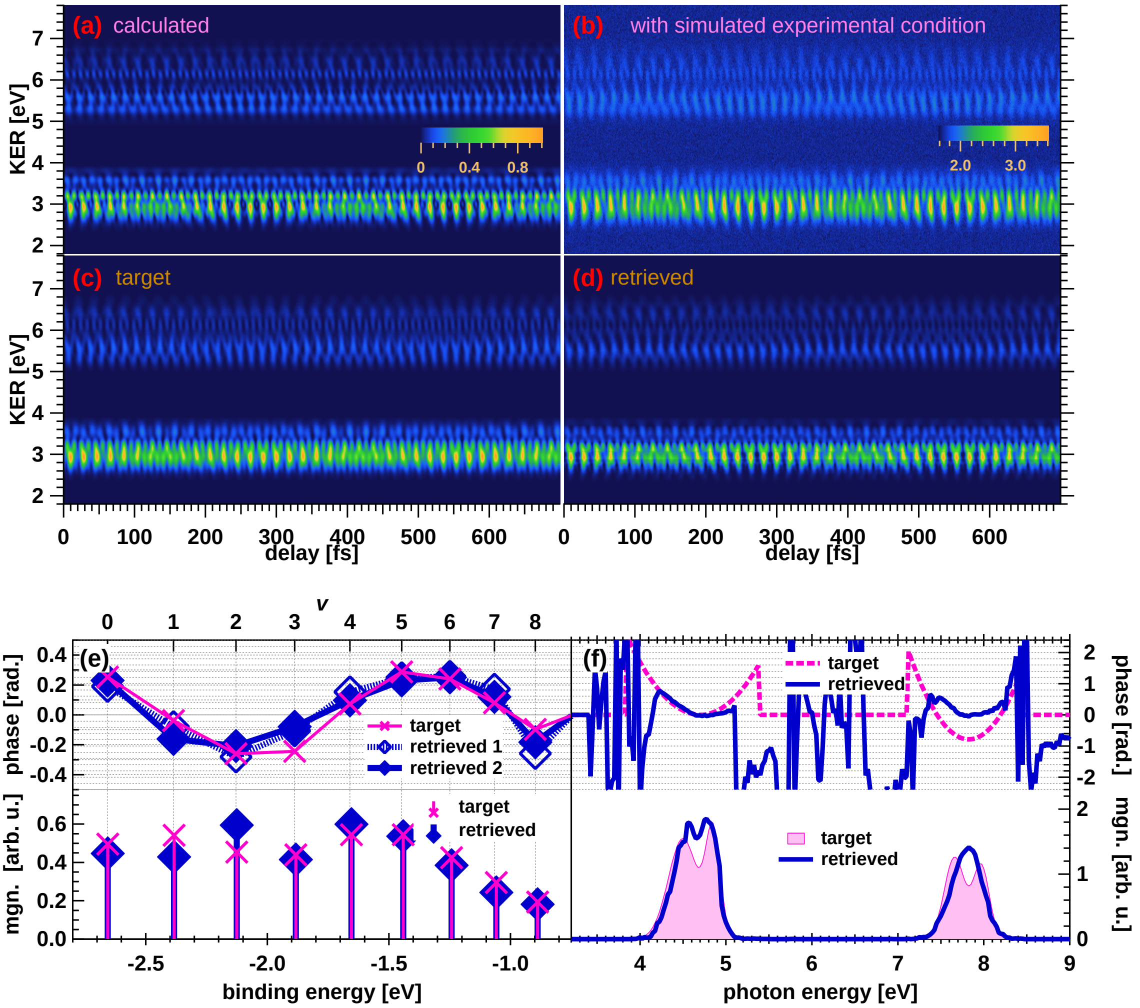

FIG. S-6. Examination of the effects of noise and degradation of the KER resolution. The amplitudes of the model vibrational wavepacket and the gate field are the same as those depicted in FIGS. S-5(c) and (d). (a) Delay-KER spectrogram calculated from $|T(\omega^u;\tau)|^2$ in Eq. (16) in the main text, which is the same as that depicted in FIG. S-5(a). The model wavepacket amplitude is depicted as crosses with bars (magnitude) and connecting lines (phase) in the bottom and top panels of FIG. S-6(e), which is the same as that depicted in FIG. S-5(c). (b) Spectrogram simulating experimental conditions, which is generated from the spectrogram in FIG. S-6(a) by applying the same procedures as that described in the legend of FIG. S-3(b). (c) Target spectrogram of MW-FROG, which is obtained by bandpass filtering of the spectrogram shown in FIG. S-6(b). (d) Spectrogram retrieved with the MW-FROG algorithm. The functional error $\Delta_{\rm rms}$, defined in the main text, is 14.7%. The convergence parameter R is 1.1, which satisfies the convergence criterion. (e) Magnitude and phase of the wavepacket amplitude a_v. The magnitude and phase retrieved from the spectrogram in FIG. S-6(c) are shown as solid squares with bars (magnitude) and connecting lines (phase) in the bottom and top panels, respectively. The phases depicted as hollow squares are obtained by the optimization of a_v and have an r.m.s. phase error of 87 mrad, while those depicted by solid squares are obtained by optimization of the polynomial expansion coefficients of the phase and have an r.m.s. phase error of 80 mrad. The phase errors are comparable to those in the retrieved phases shown in FIG. 6(c) in the main text. (f) Magnitude and phase of the gate field $\widetilde{G}(\Omega)$. We depict the magnitude and phase of the target gate field as the shaded area in the bottom panel and the dashed curve in the top panel, respectively. The solid curves in the bottom and top panels are the retrieved magnitude and phase, respectively. The double-peak features of the magnitude in both photon energy regions of the third and fifth harmonic components are blurred owing to the deterioration of the KER resolution. Nevertheless, the phases in both photon energy regions of the third and fifth harmonic components exhibit quadratic changes similar to those applied to the target phase of the gate field. The constant offset, $-\pi/4$, in the photon energy region of the fifth harmonic component is not reproduced in the retrieved phase. Thus, the MW-FROG algorithm is insensitive to the offset time of the pulse train in the train envelope. In other words, we do not need to consider the change of the offset time of the pulse train in the train envelope to retrieve the phase of the vibrational wavepacket amplitude.

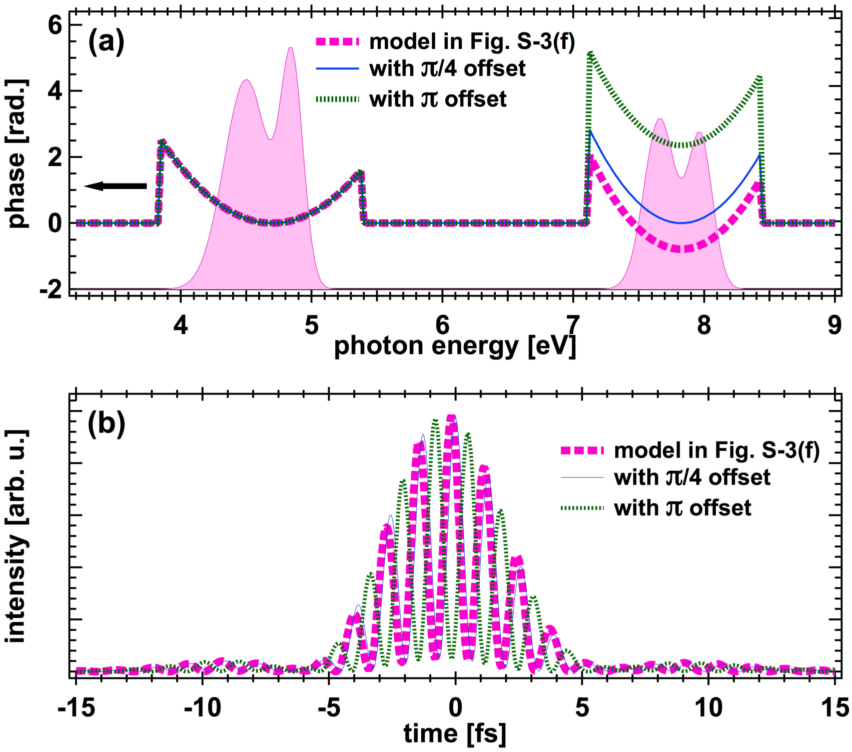

FIG. S-7. Comparison of the temporal shapes of the gate pulse train with three different phase offsets in the photon energy region of the fifth harmonic component. (a) Spectral phase of the model gate field shown in FIG. S-5(d) (dashed curve), spectral phase with an additional phase offset of $\pi/4$ (thin sold curve), and spectral phase with an additional phase offset of π (dotted curve) with respect to the dashed curve. The spectral magnitude is depicted as the shaded area. (b) Temporal profiles of the gate pulse obtained from the magnitude square of the inverse Fourier transform of the common spectral magnitude with the three spectral phases depicted in FIG. S-7(a). The offset time of the pulse train in the train envelope alters with the phase offset.
